# Longitudinal whole-genome based comparison of carriage and infection associated *Staphylococcus aureus* in northern Australian dialysis clinics

**DOI:** 10.1371/journal.pone.0245790

**Published:** 2021-02-05

**Authors:** Deborah C. Holt, Tegan M. Harris, Jaquelyne T. Hughes, Rachael Lilliebridge, David Croker, Sian Graham, Heather Hall, Judith Wilson, Steven Y. C. Tong, Phillip M. Giffard

**Affiliations:** 1 Global and Tropical Health Division, Menzies School of Health Research, Charles Darwin University, Darwin, Australia; 2 College of Health and Human Sciences, Charles Darwin University, Darwin, Northern Territory, Australia; 3 Wellbeing and Preventable Chronic Diseases Division, Menzies School of Health Research, Charles Darwin University, Darwin, Northern Territory, Australia; 4 Division of Medicine, Department of Nephrology, Royal Darwin Hospital, Darwin, Northern Territory, Australia; 5 Northern Territory Department of Health, Renal Services, Darwin, Northern Territory, Australia; 6 Victorian Infectious Diseases Service, The Royal Melbourne Hospital, and Doherty Department University of Melbourne, at the Peter Doherty Institute for Infection and Immunity, Melbourne, Victoria, Australia; Instituto de Technologia Quimica e Biologica, PORTUGAL

## Abstract

**Background:**

The study objective was to reveal reservoirs potentially leading to *Staphylococcus aureus* infections in haemodialysis clinic clients in the tropical north of the Australian Northern Territory (NT). This client population are primarily Aboriginal Australians who have a greater burden of ill health than other Australians. Reservoir identification will enhance infection control in this client group, including informing potential *S*. *aureus* decolonisation strategies.

**Methods and findings:**

The study participants were 83 clients of four haemodialysis clinics in the Darwin region of the NT, and 46 clinical staff and researchers who had contact with the clinic clients. The study design was longitudinal, encompassing swabbing of anatomical sites at two month intervals to yield carriage isolates, and also progressive collection of infection isolates. Swab sampling was performed for all participants, and infection isolates collected for dialysis clients only. Analysis was based on the comparison of 139 carriage isolates and 27 infection isolates using whole genome sequencing. Genome comparisons were based on of 20,651 genome-wide orthologous SNPs, presence/absence of the *mecA* and *pvl* genes, and inferred multilocus sequence type and clonal complex. Pairs of genomes meeting the definition of “not discriminated” were classed as defining potential transmission events. The primary outcome was instances of potential transmission between a carriage site other than a skin lesion and an infection site, in the same individual. Three such instances were identified. Two involved ST762 (CC1) PVL- MRSA, and one instance ST121 PVL+ MSSA. Three additional instances were identified where the carriage strains were derived from skin lesions. Also identified were six instances of potential transmission of a carriage strains between participants, including transmission of strains between dialysis clients and staff/researchers, and one potential transmission of a clinical strain between participants. There were frequent occurrences of longitudinal persistence of carriage strains in individual participants, and two examples of the same strain causing infection in the same participants at different times.

Strains associated with infections and skin lesions were enriched for PVL and *mecA* in comparison to strains associated with long term carriage.

**Conclusions:**

This study indicated that strains differ with respect to propensity to stably colonise sites such as the nose, and cause skin infections. PVL+ strains were associated with infection and skin lesions and were almost absent from the carriage sites. PVL- MRSA (mainly CC1) strains were associated with infection and also with potential transmission events involving carriage sites, while PVL- MSSA were frequently observed to stably colonise individuals without causing infection, and to be rarely transmitted. Current clinical guidelines for dialysis patients suggest MRSA decolonisation. Implementation in this client group may impact infections by PVL- MRSA, but may have little effect on infection by PVL+ strains. In this study, the PVL+ strains were predominant causes of infection but rarely colonised typical carriage sites such as the nose, and in the case of ST121, were MSSA. The important reservoirs for infection by PVL+ strains appeared to be prior infections.

## Introduction

*Staphylococcus aureus* is a frequent coloniser of humans and an important human pathogen. Although *S*. *aureus* is strongly associated with nosocomial infections, in recent decades diverse *S*. *aureus* lineages associated with community-onset infections have been described [1, 2]. Such lineages typically harbour the prophage that encodes the Panton-Valentine leukocidin (PVL). The PVL phage specifies a pore forming toxin that is associated with *S*. *aureus* infections causing furunculosis and necrotising pneumonia, often in disadvantaged populations [3].

It is widely accepted that colonising *S*. *aureus* constitutes a reservoir that increases the possibility of infection by autoinoculation, or transmission to others. This notion underpins the widespread practice of decolonisation, usually by nasal application of mupirocin, sometimes combined with chlorhexidine body washing [3–6]. Possible consequences of decolonisation include reduced risk of infection by autoinoculation, to broader effects on strain prevalences within health care facilities or in the general population. Most decolonisation policies are targeted at MRSA because of the specific clinical risks associated with resistance to β-lactam antibiotics. In general carriage strains are not otherwise stratified as to whether they do or do not trigger decolonisation, even though it is plausible that virulence factors such as PVL could modulate the risk posed by carriage [6].

Adults dependent on chronic maintenance haemodialysis constitute a high-risk population group for *S*. *aureus* infection [7–10]. Such clients represent a specific risk profile and infection control requirements. They are in frequent contact with both the clinical environments of satellite dialysis units and/or hospitals and the outside community, they are reliant on *in situ* vascular access, and these clients may concurrently live with advanced comorbidity including poorer immune function [11]. An extraordinarily high burden of end stage kidney disease is experienced in northern Australia, which disproportionately affects Aboriginal peoples [12, 13]. Within this region, there is also an unusually high prevalence of *S*. *aureus* infection, much of which is associated with known community acquired strains [14, 15].

A recommendation of the current Kidney Health Australia haemodialysis infection control guidelines [16] is “We suggest screening of MRSA in outpatient haemodialysis patients and subsequent infection control interventions including transmission based precautions in centres with infections related to MRSA or more than a very low colonisation rate”. *S*. *aureus* nasal decolonisation is a potential infection control intervention [9], and need not necessarily be confined to MRSA. Decolonisation procedures are inexpensive and non-invasive. However, they do have the potential to select mupirocin resistance [5, 9]. This study was designed to have the potential to inform refinement of such guidelines.

We have addressed the question as to whether there is evidence for transmission between sites that could potentially be decolonised, and sites where clinical infections have occurred. We undertook a prospective longitudinal study to identify reservoirs giving rise to *S*. *aureus* infection in adults dependent on chronic maintenance haemodialysis in the north of the Australian Northern Territory (NT). The primary objective was identification of instances where *S*. *aureus* carriage in a client potentially led to infection in the same person. The basis for the experimental design was whole genome-based comparison of carriage strains and strains derived from infection. This experimental design also allowed identification of other classes of transmission events, in particular those involving different individuals. Accordingly, surveillance of carriage strains encompassed clinical and research staff as well as dialysis clients.

## Methods

### Ethics statement

Ethical approval for the study was received from the Human Research Ethics Committee of the Northern Territory Department of Health and Menzies School of Health Research and their Aboriginal Ethics subcommittee (EC00153- approval numbers 12–1762 and 13–1943). Participants were provided with verbal and written information on the study and gave written informed consent if they wished to participate. Participants could withdraw completely from the study at any time, could decline to participate in any given round of swab collection, or could decline any particular type of swab during each swab collection round.

### Participant recruitment

The clinical phase of the study was conducted from November 2012 to December 2014 at four haemodialysis units. These provided nurse-assisted dialysis treatments, and were operated by an NT Government owned dialysis provider, the major supplier of renal care in this region. Three sites were in urban regions within 20 km of the major nephrology care hub in Darwin, and one was in a remote Indigenous community. Clients and haemodialysis clinical staff at all four sites were invited to participate in the project, as were research staff involved in the project. Participants were recruited on a rolling basis, with any new clients or staff present at a study site during each swab collection round invited to participate. Because the primary outcome of the study was identification of potential transmission events leading to infection, each pair-wise genomic comparison of isolates could be regarded as a separate experiment. Therefore, for the primary outcome, the number of participants was not a driver of the probability of statistically significant results being obtained.

### Clinical information

All clients were receiving chronic maintenance haemodialysis. Basic demographic information including date of birth and gender were collected. Date of initiation of chronic maintenance haemodialysis was confirmed from Australia and New Zealand Dialysis and Transplant Register (www.anzdata.org.au), to calculate age and duration of treated end-stage kidney disease, and to compare the client participants of the study with other clients of the renal care provider, who were non-participants in the study.

### Swab collection

Initially a small-scale pilot study at two of the urban study sites was conducted to trial the participant information and consent processes, and the swab collection protocol. This initial phase involved monthly swab collection visits at two of the study sites for three months (Rounds P1 to P3). Based on feedback from participants during this phase, the full study involved swab collection rounds every second month (Rounds 1 to 10- numbered consecutively for each participant). The additional two study sites were included as the study progressed. The study team visited the study sites and collected swabs from haemodialysis clients and clinical staff, while swabs from research staff participants were self-collected in designated clinic facilities within the research institute. For both phases of the study, contact between the study team and the Aboriginal participants was implemented by two Aboriginal Research Assistants, who also provided leadership in developing the protocols for interactions with Aboriginal participants.

At each swab collection round, swabs were requested from the nasal passage, axilla, groin, haemodialysis access site (either a vascular access site (collected from the intended needling sites on the arterio-venous fistula prior to antiseptic skin preparation) or vascular access catheter), and any other skin lesions observed. Rayon tipped cotton swabs (Copan 155CIS: Interpath, Heidelberg West, Victoria, Australia) were moistened with sterile saline and lightly rubbed on the relevant area. The swabs were then put into skim milk tryptone glucose glycerol broth, transported back to the laboratory chilled, and stored at -80°C.

### Culture of collected swabs

Stored swabs were thawed, plated onto Columbia colistin nalidixic acid (CNA) horse blood agar (Oxoid- ThermoFischer, Scorsby, Victoria, Australia), and incubated for up to 48 hours at 37°C in 5% CO_2_. Up to 5 suspected *S*. *aureus* colonies were sub-cultured for each swab and were confirmed as *S*. *aureus* by a positive reaction using a Staphytect Plus kit (Oxoid). Positive isolates were stored in tryptone soya broth (Oxoid) containing 15% glycerol at -80°C.

### Clinical isolates

Available *S*. *aureus* isolates from clinical episodes of disease were obtained for client participants through the regional hospital pathology department and were sub-cultured and stored as above. This was carried out throughout the course of the study.

### Genome analyses of *S*. *aureus* isolates

Up to two morphologically distinct stored *S*. *aureus* isolates from each swab were sub-cultured on Columbia CNA horse blood agar (Oxoid). Genomic DNA was extracted using a QIAamp DNA Mini Kit (Qiagen, Chadstone, Victoria, Australia) with the addition of lysostaphin (Sigma, Sydney, New South Wales, Australia) and whole genome sequencing was carried out on the Illumina HiSeq 2500 platform with Nextera XT library preparation and150bp paired end reads (Macrogen Inc., Seoul, South Korea). Alternatively, 2mL tryptone soy broth cultures of isolates were sent to the Australian Genome Research Facility for DNA extraction and genome sequencing carried out on the Illumina HiSeq 2500 platform with Nextera XT library preparation and 125bp paired end reads. The data for this study have been deposited in the European Nucleotide Archive (ENA) at EMBL EBI under study accession number PRJEB40888. Genome accession numbers for each sequenced isolate are given in S2 and S3 Data.

In silico multi-locus sequence typing (MLST) and identification of PVL and *mecA* genes was performed on the Illumina short read data using ARIBA v2.9.4 [17]. Genome analysed isolates were assigned to a “strain”, comprised of the inferred multi-locus sequence type (ST), the clonal complex (CC), and the presence/absence of the PVL encoding prophage and *mecA*. This CC parameter is the CC to which the ST belongs, as defined in the *S*. *aureus* PubMLST database (pubmlst.org/saureus). For STs not assigned to a CC in the *S*. *aureus* PubMLST database, this parameter was omitted. This strain naming procedure was devised because the MLST-based descriptions of *S*. *aureus* population structure are common and use a stable numbering convention, the presence of *mecA* is frequently a trigger for decolonisation, and PVL is robust marker of community onset lineages that are often of increased virulence.

Short read data of sequenced isolates were aligned to the genome sequence of *S*. *aureus* isolate Mu50 (NC002758.2) [18], and an orthologous SNP matrix generated using SPANDx v3.2.1 [19]. A maximum parsimony tree was built using PAUP v4.0a150 [20] and visualised using Phandango v1.3.0 [21]. For a subset of STs, the process was repeated such that all isolates of the ST were aligned to the genome sequence of a *S*. *aureus* isolate of the same ST or clonal complex (ST5- Mu50, ST72- CN1(NC_022226.1)), ST93- JKD6159 (NC_017338.1), ST762- MW2 (NC_003923), ST789- NCTC8325 (NC_007795.1).

There were instances in which additional *S*. *aureus* colonies from swabs were purified and subjected to genome analysis. This was prompted by instances of where a low resolution genotyping method (Minim typing [22]) revealed different genotypes from a skin infection clinical isolate and a skin lesion “carriage” isolate from the same participant, or where a different strain was identified in a series of similar strains associated with longitudinal carriage in an individual. This occurred with seven dialysis client participants. The relevant isolates are flagged as such in the Results.

### Definition of potential transmission events

To investigate possible transmission events, instances of isolates with “non-discriminated” genome sequences were identified, with the rationale that these represent potential transmission events. The criterion for classifying two or more genomes as non-discriminated was developed as follows. As a reference point, isolates from the two cases indicative of longitudinal carriage of the same isolate in individual participants were assumed to represent single clones. The first case was a staff participant (1046) who was colonised in the nose with ST12 isolates at all sampled time points over an 18 month period (S5 Data—Group 28). These was a single SNP in one genome of these nine isolates from eight time points. The second case was another staff participant (1045) who was colonised in the nose with ST5 isolates at all but one sampled time points over a 16 month period (S5 Data—Group 7). There was a maximum of 5 SNPs between any pair of the seven isolates from six time points. On this basis, the genomes of sequenced isolates were classed as non-discriminated if they differed by 5 SNPs or less in the orthologous SNP matrix.

Detailed studies of potential transmission events of a single ST of *S*. *aureus* have suggested 30 SNPs [23] and 39 SNPs [24] as thresholds for genome identity when closely related isolates are compared. As we undertook alignment of all of the sequenced isolates from this study to a single reference genome of ST5, we compared our empirically calculated cut off value of ≤5 SNPs for genome non-discrimination from the full orthologous SNP matrix, to more detailed single ST analyses that more closely reflect the published studies. All isolates of STs 5, 72, 93, 762, and 789 were individually aligned with a reference genome sequence of the same ST or CC, and an ST specific orthologous SNP matrix was produced as described above. These individual ST specific orthologous SNP matrices covered a total of 14 of the non-discriminated groups of genomes defined using the empirically determined ≤5 SNP cut off. Using a SNP cut off of ≤30 SNPs with the ST specific orthologous SNP matrices, the same 14 groups containing the same non-discriminated genomes were defined. There was one instance in which two discriminated groups of ST762 genomes defined one pair of genomes that had a pairwise SNP difference of 29 SNPs. As the pairwise SNP differences between all other pairs of isolates between these groups ranged from 31 to 47, we continued to define these as two separate groups (S5 Data- Groups 1 and 2). The maximum number of pairwise SNPs between all pairs of genomes in each of the defined non-discriminated groups is given in S5 Data for the all isolate orthologous SNP matrix and for the ST specific orthologous SNP matrices where performed. This analysis indicates that the empirically calculated cut off of ≤5 SNPs for the all isolate orthologous SNP matrix provides essentially identical results to analysis of the individual STs using previously published SNP thresholds.

Potential transmission events were subdivided according to whether the carriage isolate had been obtained from a skin lesion, or from another site. This was because genome identity between clinical isolates and skin lesion “carriage” isolates may be less remarkable than genome identity between clinical isolates and other carriage isolates, because of the possibility of a clinical isolate arising from the same skin lesion that was swabbed in the course of our carriage study.

### Statistical methods

Frequencies were compared using the Fisher exact probability test, using the on-line facility: http://vassarstats.net/odds2x2.html.

## Results

### Study participants

A total of 129 individuals consented to participate in the study and underwent at least one round of swab collection. Of these, 83 (64%) were dialysis clients (“clients”), and 46 (36%) were clinical or research staff (“staff”). Of the 65 client participants who were registered with the Australia and New Zealand Dialysis and Transplant Register (ANZDATA), their demographic and clinical characteristics were similar to the characteristics of 294 other prevalent clients receiving chronic maintenance haemodialysis care by the same health care provider who were also registered with ANZDATA (S1 Data).

### Recovery of carriage isolates

Participants were enrolled on a rolling basis from November 2012 to December 2014, and were approached for swab collection monthly during an initial three month pilot study (Rounds P1 to P3), then every two months for the remainder of the study (Rounds 1 to 10). A total of 580 individual rounds of swab collection were undertaken, with a mean of 4.5 rounds of swab collection per participant (clients: mean 4.2, range 1–10; staff: mean 5.0, range 1–12) (Table 1). A total of 2098 swabs were collected from participants, from the nasal passage, axilla, groin, dialysis vascular access site, and any identified skin lesions.

**Table 1:** Participant recruitment, swab sampling, and *S*. *aureus* recovery.

	All	*S*. *aureus*	Clients	*S*. *aureus*	Staff	*S*. *aureus*
	Total	positive	Total	positive	Total	positive
Participants	129		83 (64.3%)		46 (35.7%)	
Total swabs						
Swabs	2098	127 (6.1%)	1401	85 (6.1%)	697	42 (6.0%)
Participants	129	51 (39.5%)	83	38 (45.8%)	46	13 (28.3%)
Nasal						
Swabs	579	70 (12.4%)	348	39 (11.2%)	231	31 (13.4%)
Participants	129	35 (27.1%)	83	24 (28.9%)	46	11 (23.9%)
Axilla						
Swabs	580	13 (2.2%)	349	10 (2.9%)	231	3 (1.3%)
Participants	129	9 (7.0%)	83	7 (8.4%)	46	2 (12.5%)
Groin						
Swabs	579	31 (5.4%)	348	23 (6.6%)	231	8 (3.9%)
Participants	129	19 (%)	83	14 (16.9%)	46	5 (10.9%)
Access site						
Swabs	316	3 (0.9%)	316	3 (0.9%)	NA	NA
Participants	79	3 (3.8%)	79	3 (3.8%)	NA	NA
Catheter						
Swabs	14	1 (7.1%)	14	1 (7.1%)	NA	NA
Participants	8	1 (12.5%)	8	1 (12.5%)	NA	NA
Lesion						
Swabs	30	9 (30.0%)	26	9 (34.6%)	4	0 (0%)
Participants	24	8 (33.3%)	20	8 (40%)	4	0 (0%)

NA: Not applicable.

Full information on all carriage isolates recovered can be found in S2 Data.

*S*. *aureus* was recovered from 127 of the collected swabs (6.1%), with the overall rate of *S*. *aureus* positive swabs between the client and staff participants being similar (6.1% for clients, 6.0% for staff). Fifty-one of the 129 participants (39.5%) had at least one *S*. *aureus* positive swab during the course of the project (38/83 clients (45.8%), 13/46 staff (28.3%)) (Table 1). *S*. *aureus* was most commonly recovered from nasal swabs (11.2% of client nasal swabs, 13.4% of staff nasal swabs), followed by the groin. In contrast *S*. *aureus* was recovered from 30% (9/30) of skin lesion swabs, all from clients. Recovery of *S*. *aureus* from swabs of dialysis vascular access sites (needling sites and catheters) was 1.2% (4/330) (Table 1). Full information on the carriage isolates recovered is given in S2 Data.

### Clinical isolates

In the course of the study, 28 clinical isolates from 13 client participants were obtained from the regional hospital pathology department. It was notable that 11 of the isolates were from one participant (participant 1027). Twenty-three of the isolates were from skin infections, two were from dialysis access sites, and two were from blood cultures (S3 Data).

### Whole genome sequence analyses

Whole genome sequence data was successfully obtained from 139 carriage isolates and 27 of the 28 clinical isolates. Genome analysed isolates were assigned to a “strain”, comprised of the inferred multi-locus sequence type (ST), the clonal complex (CC) if applicable, and the presence/absence of *mecA* and the PVL encoding prophage (S2 Data for carriage isolates and S3 Data for clinical isolates). Four of the carriage isolates were identified as ST1223 or ST1850 which are *Staphylococcus argenteus* sequence types [25]. A maximum parsimony tree based on 20,651 orthologous SNPs between all the sequenced isolates is shown in S1 Fig (orthologous SNP matrix given in S4 Data). All groups of genomes considered “non- discriminated” (differing by ≤5 SNPs based on the all isolate orthologous SNP matrix which we calculated to be comparable to a published threshold of ≤30 SNPs for analysis of closely related isolates within a single ST—see Methods) were identified (S5 Data).

### Potential transmission from carriage site to infection site in the same person

The primary objective of the study was identification of instances where *S*. *aureus* carriage in a client participant potentially led to infection in the same person. There were six potential instances of transmission from a carriage site to an infection site in the same dialysis client. Three of these instances encompassed isolates from carriage sites that were not skin lesions.

**Fig 1:**
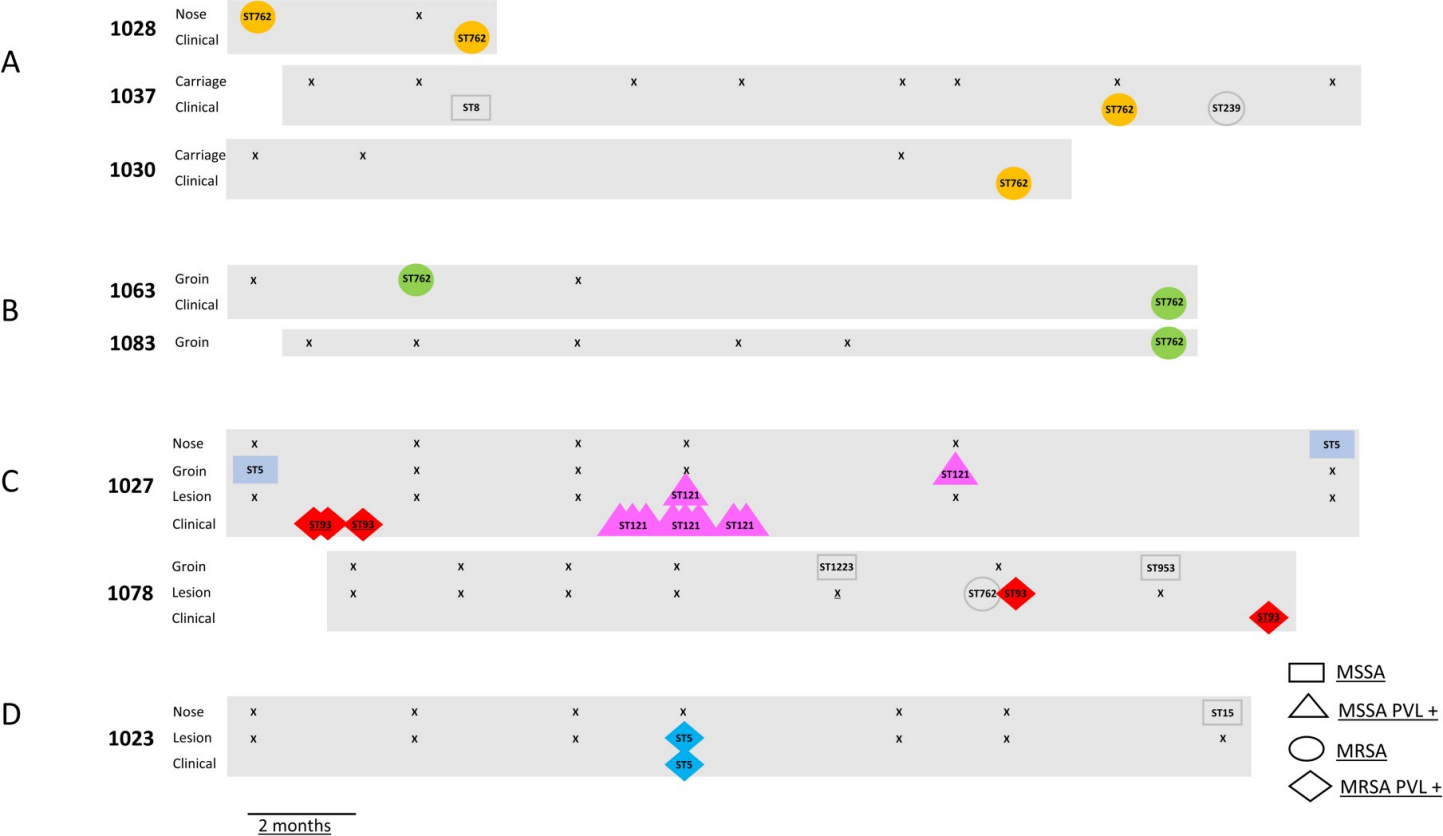
Potential transmission events between carriage sites and sites giving rise to clinical isolates. The numbers denote participants. The sites of isolation are indicated. “Lesion” means a skin lesion that was swabbed by the research team, while “clinical” denotes isolates obtained from the regional hospital pathology department. The strains are defined by the ST within the shape, and the shape itself which indicates *mecA* and PVL presence or absence. Where the same strains as defined by MLST, mecA and PVL have the same colour, they have “not discriminated” genomes. “x” represents a swabbing procedure that yielded no *S*. *aureus* or *S*. *argenteus* isolates. The graphic is time calibrated, with isolates aligned to the month of isolation.

First, non-discriminated genomes were derived from a nasal carriage isolate and an umbilicus clinical isolate from the same client participant (1028), with the nasal isolate obtained four months before the clinical isolate (Fig 1B, S5 Data- Group 1). The genomes defined an ST762 (CC1) PVL- MRSA strain. A clinical foot wound isolate and an abdominal lesion isolate from client participants 1030 and 1037 respectively, were not discriminated from the participant 1028 isolates. These were obtained 10 and 12 months respectively after the participant 1028 clinical isolate.

Second, non-discriminated genomes were derived from a clinical isolate (finger lesion) and a groin isolate in the same client participant (1063) obtained 14 months prior to the clinical isolate (Fig 1B, S5 Data- Group 2). These isolates were also ST762 (CC1) PVL- MRSA, but were at least 6 SNPs different from the three isolates described in the previous paragraph. A groin carriage isolate from a client participant (1083) from the same clinic was not discriminated from the participant 1063 isolates. This was obtained in the same month as the participant 1063 clinical isolate.

The third instance involved ST121 PVL+ MSSA isolates. Non-discriminated genomes were derived from eight clinical isolates from client participant (1027) within a three month period (Fig 1C, S5 Data- Group 3). The infections were in various anatomical locations including groin, knee, and inguinal canal. A non-discriminated genome was derived from a groin carriage isolate from the same participant, four months after the last clinical isolate was obtained. Also, a skin lesion carriage isolate with a non-discriminated genome was obtained from the same participant, within the same three month period as the cluster of clinical isolates. As shown in Fig 1C, participant 1027 also gave rise to ST5 (CC5) PVL- MSSA groin and nasal isolates with non-discriminated genomes, 20 months apart (S5 Data- Group 14). Within these 20 months, multiple clinical isolates of ST93 PVL+ MRSA (three non-discriminated genomes (S5 Data- Group 6), see also below) and the ST121 PVL+ MSSA clinical isolates described above were obtained. No additional carriage isolates were detected, and there was no evidence for infection by the ST5 (CC5) PVL- MSSA carriage strain.

There were also three instances of non-discriminated genomes being derived from clinical isolates and skin lesion-derived “carriage” isolates in the same participant. The first instance involved ST121 PVL+ MSSA isolates derived from participant 1027 (described above- Fig 1C), which encompassed genome identity between a clinical isolate and skin lesion carriage isolate. The second instance involved ST5 (CC5) PVL+ MRSA. At approximately the same time, isolates with non-discriminated genomes were derived from a clinical isolate (boil) and a skin lesion carriage isolates from client participant 1023. (Fig 1D, S5 Data- Group 5). The third instance involved ST93 PVL+ MRSA isolates from client participant 1078. Two skin lesion carriage isolates with non-discriminated genomes were derived from a thigh lesion on the same day. A third isolate with a non-discriminated genome was a clinical isolate, recorded as from a “flank wound”, derived less than five months later (S5 Data- Group 6). These three ST93 PVL- MRSA isolates were non-discriminated from the participant 1027 ST93 PVL- MRSA isolates discussed above (Fig 1C, S5 Data- Group 6). Participant 1078 also gave rise to ST1223 (*S*. *argenteus*) PVL- MSSA and ST953 PVL- MSSA groin carriage isolates six months apart, and a ST762 (CC1) PVL- MRSA isolate that was derived from the skin lesion at the same time as the ST93 PVL+ MRSA lesion isolates (Fig 1C).

### Identification of other potential transmission events

**Fig 2:**
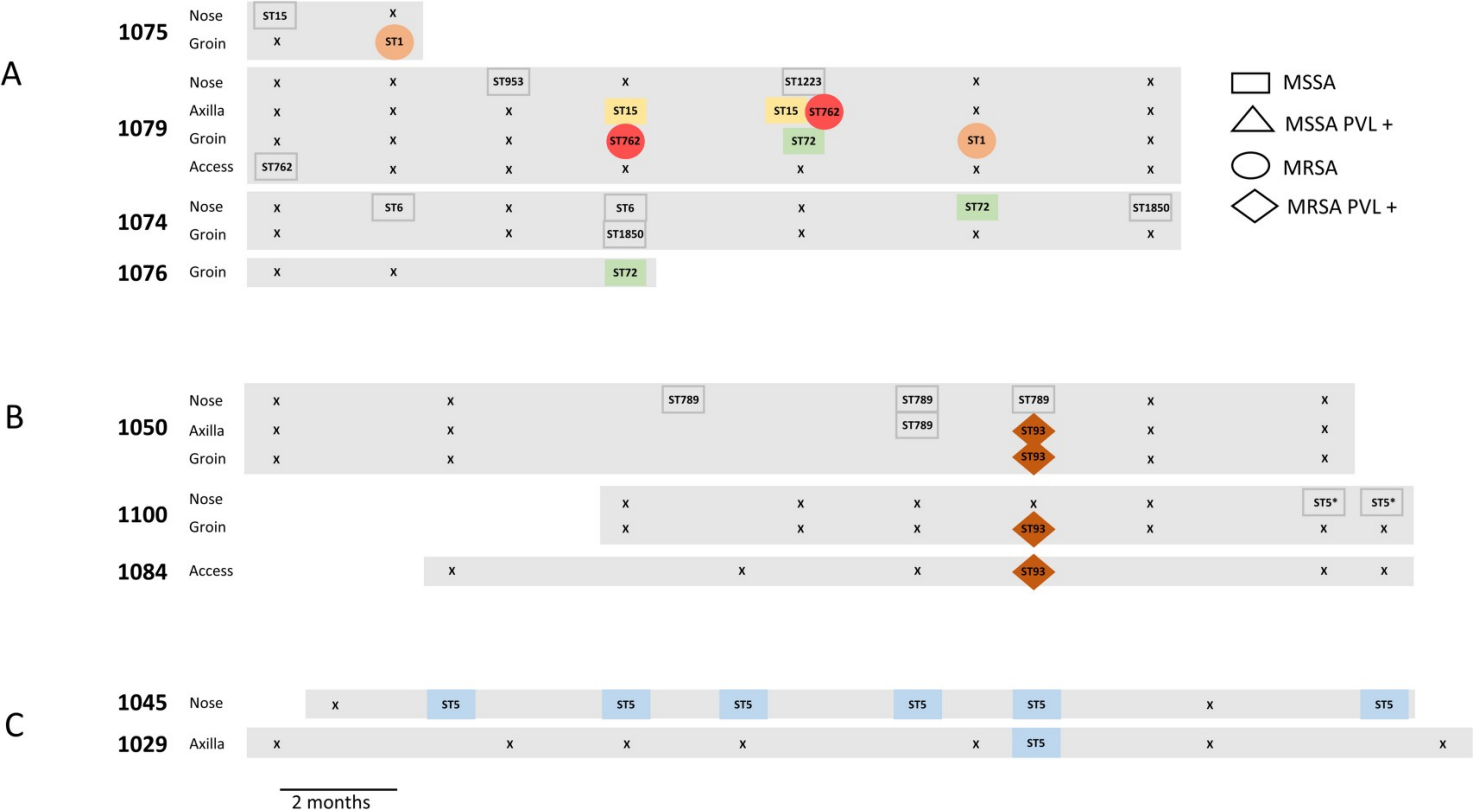
Potential transmission events between carriage sites. The numbers denote participants. The sites of isolation are indicated, with “access” denoting “access site”. The strains are defined by the ST within the shape, and the shape itself which indicates *mecA* and PVL presence or absence. Where the same strains as defined by MLST, mecA and PVL have the same colour, they have “not discriminated” genomes. “x” represents a swabbing procedure that yielded no *S*. *aureus* or *S*. *argenteus* isolates. The graphic is time calibrated, with isolates aligned to the month of isolation.

Apart from addressing our central questions regarding carriage leading to a clinical infection, the genome analyses provided broader insight into colonisation and transmission dynamics. There were six instances of potential transmission of carriage strains between individuals (Fig 2). Three of these involved MRSA strains (ST762 (CC1), ST1 (CC1), and ST93 PVL+). The instance involving ST93 reflected potential transmission between two clinical staff participants, and a dialysis client participant.

Two of these instances encompassed four participants linked by a complex pattern of potential transmission events (Fig 2A). All participants were clients of a dialysis clinic geographically separate from the other clinics in the study. Non-discriminated ST72 (CC8) PVL- MSSA genomes were identified in carriage isolates from client participants 1079, 1074, and 1076 over a period of six months (S5 Data- Group 8). Non-discriminated ST1 (CC1) PVL- MRSA genomes were identified in groin carriage isolates from participants 1079 and 1075 ten months apart, and an additional non-discriminated genome was subsequently found in a nasal isolate from a client participant (1104) outside this group of four individuals (S5 Data- Group 10). Participant 1079 was also colonised in the groin and axilla by an ST762 (CC1) PVL- MRSA over a period of three months (two isolates with non-discriminated genomes, S5 Data- Group 12), and six months before that with an ST762 PVL- MSSA isolate derived from an access site. Further, this participant also gave rise to non-discriminated ST15 (CC15) PVL- MSSA axilla isolates three months apart (S5 Data- Group 27), and ST953 (CC97) PVL- MSSA and ST1223 (*S*. *argenteus*) PVL- MSSA nasal isolates were also recovered from this participant. Participant 1075 also yielded an ST15 (CC15) PVL- MSSA nasal carriage isolate with a non-discriminated genome from a nasal carriage isolate five months later in another client participant (1096) outside this group (S5 Data- Group 35), however these genomes could be discriminated from the ST15 genomes mentioned above.

Two of the remaining four instances were of potential transmission between staff participants and client participants. In one, ST93 PVL+ MRSA isolates with non-discriminated genomes were derived from the axilla and groin of a staff participant (1050), the groin of another staff participant (1100), and the access site of a client participant (1084), all at the same time point (Fig 2B, S5 Data- Group 11). In the other instance, ST5 (CC5) PVL- MSSA isolates with non-discriminated genomes were recovered on six occasions over a 16 month period from the nose of a staff participant (1045), and on one occasion from the axilla of a client participant (1029) in the same month as one of staff participant’s isolates (Fig 2C, S5 Data- Group 7). A further instance is potential transmission between client participants 1083 and 1063 of an ST762 (CC1) PVL- MRSA strain. Participant 1063 had a groin carriage isolate which preceded a clinical infection isolate (Fig 1B, S5 Data- Group 2), in the same month as participant 1083’s groin carriage isolate. The final instance is potential transmission between client participants 1025 and 1078 of an ST953 PVL- MSSA as groin carriage isolates ten months apart (S5 Data- Group 9).

### Identification of persistent carriage

The genome analysis data also demonstrated that persistence of strains in individual participants was a frequent occurrence. This was observed with the same and with different carriage sites. Isolates with non-discriminated genomes obtained at different times from the same carriage site in a participant were exclusively PVL- MSSA strains. These were ST5 (CC5), ST5 SLV (CC5), ST88, ST101, ST789 (CC8), ST45 (CC45), ST1 (CC1), ST15 (CC15), ST12, ST72 (CC8), ST97 (CC97), ST834 (CC1), and ST8 (CC8) (S5 Data). Instances where isolates with non-discriminated genomes were obtained from different carriage sites in the same participant involved ST121 PVL+ MSSA (Fig 1C), ST78 PVL- MSSA, ST93 PVL+ MRSA (Fig 1C), ST762 (CC1) PVL- MRSA (Fig 2A), ST5 SLV (CC5) PVL- MSSA, ST5 (CC5) PVL- MSSA, ST6 (CC5) PVL- MSSA, ST88 PVL- MSSA, ST101 PVL- MSSA, and ST789 (CC8) PVL- MSSA (S5 Data). For the two instances involving PVL+ strains, one of the carriage isolates was from a skin lesion, whereas the other instances involved non-skin lesion carriage sites.

### Distributions of mecA and the PVL phage

Initial examination of the data suggested that the clinical isolates were enriched for PVL+ strains. In order to circumvent confounding effects of long-term carriage, and/or the derivation of multiple clinical isolates from the same infectious process, we determined the unique instances of participant-strain pairs. It can be seen in Table 2 and S6 Data, that clinical strains are more likely to be PVL+ than carriage strains. This analysis did not take into account that a large proportion of the PVL+ carriage isolates are obtained from skin lesions, so their status as carriage isolates is debatable. If carriage isolates obtained from skin lesions are excluded, then the correlation between being a clinical isolate and PVL+ is stronger, with a p value of 0.0008 rather than 0.003. A similar observation was made with *mecA*, with clinical strains more likely to be MRSA than carriage strains (Table 3). The corollary of this is that carriage isolates are more likely to be PVL- MSSA than clinical isolates. This association has a p value of 0.0001, irrespective of whether skin lesion “carriage” strains are included.

**Table 2:** Comparison of carriage and clinical isolates regarding PVL and *mecA* status.

	Carriage	Clinical	p	95% CI Odds Ratio
**All isolates**				
Unique participant-strain pairs	75	16		
Number of participants	51	13		
Number of strains	19	9		
PVL- MSSA (%)	54 (72)	3 (18.8)	0.00001*	0.019–0.276
PVL+ MSSA (%)	2 (2.7)	3 (18.8)	0.054	1.056–44.37
PVL- MRSA (%)	12 (16)	6 (37.5)	0.059	0.963–10.31
PLV+ MRSA (%)	7 (9.3)	4 (25)	0.098	0.820–12.78
Total PVL+ (%)	9 (12)	7 (43.8)	0.0064*	1.70–19.10
Total MRSA (%)	19 (25.3)	10 (62.5)	0.0057*	1.57–15.33
**PVL+ MRSA isolates omitted (68, 12)**				
Total PVL+ (%)	2 (2.9)	2 (16.7)	0.105	0.833–52.29
Total MRSA (%)	12 (17.6)	6 (50)	0.023	1.282–16.99

The omission of the PVL+ MRSA isolates in the bottom two rows was to test for independent effects of PVL and *mecA*.

**Table 3:** Comparison of carriage and clinical isolates regarding PVL and *mecA* status, excluding “carriage isolates”derived from skin lesions.

	Carriage	Clinical	p	95% CI Odds Ratio
**All isolates**				
Unique participant-strain pairs	65	16		
Number of participants		13		
Number of strains		9		
PVL- MSSA (%)	48 (73.8)	3 (18.8)	7.8 X 10^−6^*	0.017–0.257
PVL+ MSSA (%)	1 (1.5)	3 (18.8)	0.035	1.169–123.16
PVL- MRSA (%)	11 (16.9)	6 (37.5)	0.075	0.885–9.799
PLV+ MRSA (%)	5 (7.7))	4 (25)	0.070	0.935–17.11
Total PVL+ (%)	6 (9.2)	7 (43.8)	0.0028*	2.091–17.97
Total MRSA (%)	16 (24.6)	10 (62.5)	0.0055*	1.602–16.26
**PVL+ MRSA strains omitted (59 total)**				
Total PVL+ (%)	1 (1.7)	2 (16.7)	0.098	0.774–108.0
Total MRSA (%)	11 (18.6)	6 (50)	0.020	1.332–18.09

We reasoned that *mecA* and PVL are not independent parameters, because they frequently co-exist. In this study some ST93 and ST5 strains carried both markers. Accordingly, the analysis was repeated with all PVL+ MRSA strains omitted (bottom rows of Tables 2 and 3). The same associations were observed, suggesting that the PVL phage and *mecA* independently increase the probability that a strain will be associated with infection. However, with the application of a Bonferroni correction, this did not reach statistical significance (Table 3).

This analysis was extended by determining the relationship between carriage site, and PVL and *mecA* status (Table 4). Clinical isolates were included as a single additional category. Raw isolate numbers were used rather than unique participant-strain pairs. This revealed only a single PVL+ nasal carriage isolate, 1.4% of the total, in contrast to 60.7% of the clinical isolates being PVL+. It is also of interest that the proportion of PVL+ isolates in the nasal carriage isolates is less than in the groin and axilla isolates. The chi-square P-values for these differences are 0.05 and 0.021 respectively. Applying a correction for multiple testing (two tests resulting in P<0.025 as indicating significance) means that only the difference between the axilla and nasal isolates was classed as significant. A broadly similar pattern was seen with *mecA*, with the proportion of MRSA in nasal carriage isolates being lower than in clinical isolates. However, the difference between the proportions in nasal carriage and clinical isolates was less than for PVL phage, and differences between the nasal, groin, and axilla carriage sites were not significant.

**Table 4:** Association of PVL and *mecA* with carriage site.

Carriage site	Total isolates	PVL+ isolates (%)	MRSA (%)
Catheter skin exit site	4	2 (50)	2 (50)
Axilla	15	2 (13.3)	3 (20)
Groin	32	3 (9.4)	7 (21.9)
Nose	74	1 (1.4)	7 (12.2)
Skin lesion	11	4 (36.6)	4 (36.6)
Clinical isolates	28	17 (60.7)	13 (48.1)

## Discussion

To our knowledge, this is the first prospective study in which potential transmission of *S*. *aureus* from carriage to infection sites in a haemodialysis cohort and their carers has been identified using whole genome data. The principal findings were instances of transmission from carriage sites to sites of clinical infections, and potential transmission of infection and carriage strains between individuals. More frequently observed than potential transmission between individuals was the same strain isolated at different time points from one or more carriage sites in the same individual.

The isolates derived from infection were much more likely to harbour the PVL phage and *mecA* genes than carriage isolates. In particular, the prevalence of PVL+ strains amongst the nasal carriage isolates was very low, suggesting that PVL is associated with impaired ability to colonise the nose. A similar association was seen with the *mecA* gene, with enrichment of the *mecA* gene in infections derived isolates. However, in comparison to PVL, *mecA* was less depleted in carriage strains. This study encompassed carriage isolates from clinical staff and researchers who had potential contact with the participating dialysis clients. We identified no instances of possible direct transmission of a carriage strain from a staff member or researcher to an infection site in a dialysis client. However, possible transmission of a PVL+ MRSA strain to a carriage site on a client was identified.

The prevalence of *S*. *aureus* carriage in this study was low in comparison to other studies. A commonly stated figure for the prevalence of *S*. *aureus* carriage is 30%. However, there is wide variation between studies, and a trend for carriage rates in more recent studies to be lower [26]. Nevertheless, our finding that 11.2% of client nasal swabs and 13.4% of staff/researcher swabs yielded *S*. *aureus* is at the low end of the range of prevalences from other studies. Conversely, carriage of MRSA was more prevalent than is often reported. For example, collated data from colonisation studies in Oceania revealed 26% nasal swabs positive for *S*. *aureus* and 0.2% for MRSA [26], so 0.77% of the nasal isolates were MRSA. In the current study, 12% of the nasal isolates were MRSA, which equates to 1.5% of the nasal swabs yielding MRSA. While there is extensive reporting that the Australian Northern Territory population has a disproportionate burden of disease caused by MRSA [14, 15, 27, 28], little is known regarding the extent that this this is driven by MRSA carriage. One Indigenous community-based study from more than a decade ago did show significant MRSA carriage [29]. Consistent with this, our results show elevated MRSA carriage, even though the prevalence of *S*. *aureus* nasal carriage was low. Therefore, the most marked difference between our results and collated Oceania data is the 16X difference in the proportion of nasal carriage isolates that are MRSA.

The strains observed and their relative abundances were largely in accord with other studies [15, 30, 31]. The PVL phage was found in ST93, ST121, and ST5 (CC5). ST93 is a well-studied lineage originating in northern Australia, and associated with PVL carriage, frequent but not universal *mecA* carriage, and high virulence [32]. ST121 is a widely disseminated lineage also known to carry the PVL phage, but which very rarely carries *mecA* [33]. A very recent emergence of an ST5 PVL+ MRSA lineage in Australia has been documented [34]. The *mecA* gene was identified in the majority of ST93 isolates, both the PVL+ ST5 isolates, and also in ST239 (CC8) PVL- isolates, and ST1 (CC1) and ST762 (CC1) PVL- isolates. ST239 is a predominant hospital associated MRSA in Australia [31]. ST1 PVL- MRSA are well established as a cause of community onset infection, particularly in Western Australia [35, 36]. The study protocol was designed to not exclude *Staphylococcus argenteus* [25, 37]. Four carriage isolates were this species, two of which were ST1223 (one groin and one nasal isolate) and two were ST1850, (both nasal isolates). Both ST1850 isolates carried *mecA*, consistent with a previous report [15]. This shows that *S*. *argenteus* continues to circulate in the north of Australia [15]. However, none of the clinical isolates were *S*. *argenteus*. The maximum parsimony tree (S1 Fig) did not show the *S*. *argenteus* isolates as divergent from *S*. *aureus* as other studies [25, 37]. Our interpretation is that the use an *S*. *aureus* reference sequence has biased the analysis to genome regions where similarity between *S*. *aureus* and *S*. *argenteus* is higher. Successful alignment of an *S*. *argenteus*-derived short read with an *S*. *aureus* reference will be more likely in such regions.

The *S*. *aureus* strains appeared to assort into four “ecotypes”. One ecotype is defined by long term stable nasal carriage. The numerous examples of this were predominantly MSSA PVL- strains, that caused no infections and were not associated with skin lesions or access sites. The PVL+ strains defined a second ecotype. These strains were predominant as causes of skin infection, but were rare as carriage strains in the nose or on undamaged skin. Remarkably this entire study yielded just one haemodialysis client-derived PVL+ carriage isolate from a site that was not a skin lesion or an access site, and this isolate was not an MRSA. It is intriguing that PVL+ strains did colonise the groin and axillar sites in staff/researchers, and whole genome analyses indicated potential transmission of these strains to (or from) the access sites in a haemodialysis client.

A third potential ecotype is represented by PVL- MRSA within CC1, and in particular ST762. The findings with respect to ST762 (CC1) PVL- MRSA are notable. ST762 is represented in the *S*. *aureus* MLST database by a single isolate from Western Australia. Despite this, ST762 (CC1) PVL- MRSA comprise 5.8% of the carriage isolates in this study and 14.3% of the clinical isolates. This strain was also involved in both instances of potential carriage→clinical transmission within individuals where carriage preceded infection, one of the two instances of potential clinical→clinical transmission between participants (notably involving three participants), and one of the six instances of potential transmission of carriage strains between individuals. Also, the strain was recovered from participants in two of the participating clinics. The related ST1 PVL- MRSA was equally as prevalent as ST762 (CC1) PVL- MRSA amongst carriage isolates. Taken together CC1 PVL- MRSA constituted 12 of the 15 (80%) non-skin lesion PVL- MRSA carriage isolates from dialysis clients. These were from ten individuals, and long- term carriage was not observed. Interestingly only a single CC1 MRSA isolate was recovered from staff/researchers, and this was an ST1. Accordingly, this ecotype exhibits short term carriage and transmissibility amongst the haemodialysis clients and can cause infections. There was very little carriage of these strains by researchers/clinical staff.

A potential fourth ecotype is represented by ST239. ST239 is well established as a hospital-adapted MRSA [35]. The small number of ST239 isolates, and the complete absence of other known hospital adapted strains indicates that infection control measures targeting hospital adapted MRSA are successful in the participating clinics.

The implications of these results regarding the utility of decolonisation are complex. Notably, this study yielded no data to indicate that decolonisation of nasal or healthy skin carriage sites in the haemodialysis clients would have any protective effect against infection by PVL+ strains. However, we obtained some evidence that PVL+ strains are able to colonise the skin of staff and be transmitted to access sites, although we observed no infection associated with this. The PVL+ strains’ almost complete absence from the nose or on healthy skin of dialysis clients, in combination with their predominance in infection-related isolates, argues against the nose or healthy skin being a significant reservoir for infection with PVL+ strains. Conversely, carriage of CC1 PVL- MRSA in the nose and on healthy skin was observed, as was potential transmission of such strains, so carriage may therefore constitute a risk for infection and/or transmission. This suggests that infection control strategies for PVL+ strains and PVL- MRSA should differ. With PVL+ strains, it is likely that the predominant infection control risks are posed by active skin infections, with carriage by carers possibly contributing to risk. Conversely, haemodialysis client decolonisation of CC1 PVL- MRSA could conceivably reduce risk of infection and carriage. However, a caveat is that we obtained little evidence of stable carriage of CC1 PVL- MRSA strains by individuals, so the impact of decolonisation is difficult to predict.

Our study generated scant evidence to justify decolonisation of PVL- MSSA strains. We frequently observed long term stable carriage of PVL- MSSA strains in both dialysis clients, and staff/researchers. However, only three instances of infection by such strains were identified (one ST8, one ST5 and one ST30).

Kidney Health Australia haemodialysis infection control guidelines [16] state “We suggest screening of MRSA in outpatient haemodialysis clients and subsequent infection control interventions including transmission based precautions in centres with infections related to MRSA or more than a very low colonisation rate”. Our results from this cohort indicate that screening may provide useful information regarding the CC1 PVL- MRSA, but would not provide useful information regarding risk of infection with PVL+ strains. Further, 33% of the infection-associated isolates in our study were ST121 PVL+ MSSA, which would not be identified by MRSA screening. Another recommendation is that “decolonisation should be considered for colonised haemodialysis patients who have had MRSA related infections.”. Once again, this strategy could potentially impact risk of infection by CC1 PVL- MRSA, but would likely not impact infection by PVL+ strains. Decolonisation of carers may conceivably impact risk of transmission of PVL+ strains, but for this, it may be desirable for decolonisation to be triggered by both MSSA and MRSA PVL+ strains.

Our results show some consistency but also important differences with other studies. An *S*. *aureus* carriage study in an Indigenous community in Queensland, Australia [38] showed a similar association of PVL+ strains with skin infections, and their absence from nasal carriage, with a converse picture for PVL- strains. A study of Royal Marine [39] recruits in the UK showed very little carriage of PVL+ *S*. *aureus*, although there was a high prevalence of skin and soft tissue infections. However, other studies have shown high prevalence of carriage with PVL+ strains [40–43]. A whole genome-based study to attempt to identify an *S*. *aureus* carrier causing paediatric infections in a hospital revealed a PVL+ strain was being carried and transmitted by a staff member [44]. Interestingly there is a report that PVL+ strains are easier to remove from carriage sites than PVL- strains [45]. Studies similar to ours, although smaller in scale and with mainly lower resolution genotyping, showed more frequent concordance between carriage strains and infection strains than we observed [46, 47]. There have been implementations of *S*. *aureus* decolonisation in haemodialysis clients. A New Zealand based study [48] targeted both MSSA and MRSA and showed that decolonisation persists for some weeks. This study did not address the burden of infection. A UK based study had a similar design and outcomes [49], and in addition demonstrated a correlation between decolonisation failure and risk of bacteraemia. The authors recommended screening and “aggressive attempts at decolonisation” aimed at all *S*. *aureus*.

The principle limitation of this study is that it is impossible to be certain that pairs of isolates with genomes that are “not discriminated” have arisen from single transmission events, even when epidemiological evidence suggests that this is the case. What this study has done is place upper boundaries on the frequencies of transmission between sampled sites, and stable carriage of clones over time by individuals. The rarity of potential transmission events compared with the frequent observation of stable carriage does suggest that the genome analysis had sufficient resolving power to yield meaningful conclusions. Another limitation is that we did not systematically search for multiple strains. On a small number of occasions we identified mixed strains in skin lesions. We cannot rule out that mixed strains have resulted in instances of potential transmission being missed. However once again the very frequent observation of stable carriage of strains over time suggests that mixed strains at typical carriage sites is not common.

In conclusion, whole genome analysis of *S*. *aureus* associated with patients and staff from haemodialysis clinics in the tropical north or Australia revealed frequent long-term stable carriage by diverse PVL- MSSA, frequent infections with little evidence of a carriage reservoir(s) by PVL+ strains (ST93, CC121, CC5), and unstable carriage, likely frequent transmission, and significant burden of infection from PVL- MRSA in CC1 (ST762 and ST1). The results do not suggest that any decolonisation protocol would have a large impact on the burden of *S*. *aureus* infection in patients similar to this cohort. However, decolonisation triggered by MRSA strains in the nose or healthy skin of clients, or PVL+ strains in the nose or healthy skin of carers could conceivably reduce infection risk.

## Supporting information

S1 Fig Maximum parsimony tree of genome sequenced carriage and clinical isolates. The maximum parsimony tree is based on 20,651 orthologous SNPs generated from alignment of all sequenced isolates with *S*. *aureus* isolate Mu50. Sequenced isolates are named STR (study name) followed by a unique four digit participant number. Clinical isolates are followed by the date of isolation with an additional number if more than one isolate was recovered on the same day. Carriage isolates are followed by three digits- the first is the round number, the second is the site of isolation (1 = nose, 2 = axilla, 3 = access site, 4 = catheter, 5 = groin, 6 = skin lesion), the third is the unique isolate number.
(TIF)

S1 Data Comparison of the characteristics of client study participants with other dialysis clients of the same dialysis provider, registered with Australia and New Zealand Dialysis and Transplant Register (ANZDATA). (DOCX)

S2 Data Carriage isolates recovered. Details of recovered carriage isolates including site of isolation, mecA and PVL status, sequence type (ST), and alignment metrics.
(XLSX)

S3 Data Clinical isolates recovered. Details of recovered carriage isolates including site of isolation, mecA and PVL status, sequence type (ST), and alignment metrics.
(XLSX)

S4 Data Orthologous SNP matrix. The orthologous SNP matrix in NEXUS format representing 20,651 informative SNPs based on alignment of the sequenced isolates to *S*. *aureus* isolate Mu50.
(NEX)

S5 Data Groups of non-discriminated genomes. (DOCX)

S6 Data Unique participant isolate pairs. C: Client participant; S: Staff participant.
(DOCX)
