# Supplementary material for: Longitudinal whole-genome based comparison of carriage and infection associated Staphylococcus aureus in northern Australian dialysis clinics
Source: PLoS One. 2021 Feb 5;16(2):e0245790. doi: 10.1371/journal.pone.0245790 (PMC7864423; doi:10.1371/journal.pone.0245790)
Supplement: S1 Data — (DOCX) [file pone.0245790.s002.docx]

**S1 Data. Comparison of the characteristics of client study participants with other dialysis clients of the same dialysis provider, registered with Australia and New Zealand Dialysis and Transplant Register (ANZDATA)**

These data are relevant to the question as to whether the study participants who were dialysis clients, were typical of clients of the dialysis provider. Of the 83 participants in the study who were clients of the dialysis provider, we were able to locate corresponding data in ANZDATA for 65. We also located data in ANZDATA concerning 294 individuals who were clients of the dialysis provider during the time of the study, and who were not study participants. These participant and non-participant cohorts were compared with respect to vascular access type, diabetes burden, age at recruitment into the study, and length of time on dialysis at the time of recruitment into the study.

**Vascular Access Type**

|  | **Participants (%)** | **Non-participants (%)** |
| --- | --- | --- |
| Unknown | 0 (0%) | 15 (5.1%) |
| Haemodiafiltration | 0 (0%) | 2 (0.7%) |
| Haemodialysis-hollow fibre dialysers | 57 (87.7%) | 218 (74.1%) |
| Peritoneal - automated (apd) | 3 (4.6%) | 17 (5.8%) |
| Peritoneal - continuous ambulatory (capd) | 5 (7.7%) | 42 (14.3%) |
| **Total** | **65 (100%)** | **294 (100%)** |

**Diabetes burden**

|  | **Participants (%)** | **Non-Participants (%)** |
| --- | --- | --- |
| Not diagnosed with diabetes | 26 (40.0%) | 123 (41.8%) |
| Type I insulin-dependent | 1 (1.6%) | 7 (2.4%) |
| Type II non-insulin-dependent | 32 (49.2%) | 131 (44.5%) |
| Type II insulin-requiring | 6 (9.2%) | 33 (11.3%) |
| **Total** | **65 (100%)** | **294 (100%)** |

**Age at commencement of the study**

**Length of time on dialysis at commencement of the study**

Note “0” years on dialysis refers to dialysis clients who commenced dialysis during the course of the study.
