# Supplementary material for: Longitudinal whole-genome based comparison of carriage and infection associated Staphylococcus aureus in northern Australian dialysis clinics
Source: PLoS One. 2021 Feb 5;16(2):e0245790. doi: 10.1371/journal.pone.0245790 (PMC7864423; doi:10.1371/journal.pone.0245790)
Supplement: S5 Data — (DOCX) [file pone.0245790.s006.docx]

**S5 Data. Groups of non-discriminated genomes.**

Isolates were considered to be not discriminated from each other if they differed by ≤5 SNPs in the orthologous SNP matrix of 20,651 variable nucleotide positions (see Methods). The relationships between the non-discriminated isolates were classified as follows:

1. Identification of non-discriminated genome sequences in carriage isolates at the same site, in the same participant.
2. Identification of non-discriminated genome sequences in carriage isolates from different sites, in the same participant.
3. Identification of non-discriminated genome sequences in carriage isolates, from different participants.
4. Identification of non-discriminated genome sequences in a clinical isolates and “carriage’ skin lesion isolates, in the same participant.
5. Identification of non-discriminated genome sequences in clinical isolates and carriage isolates not including “carriage” skin lesion isolates, in the same participant.
6. Identification of non-discriminated genome sequences in different clinical isolates, from the same participant.
7. Identification of non-discriminated genome sequences in clinical isolates, from different participants.

“Carriage” skin lesion isolates are defined as isolates that were recovered from skin lesions that were swabbed as part of the routine carriage swabbing activities.

| **Group number** | **Sequence type** | **PVL** | ***mecA*** | **Number of isolates** | **Maximum SNPs*** | **Description** |
| --- | --- | --- | --- | --- | --- | --- |
| 1 | ST762  (CC1) | Neg | Pos | 4 | 7  (24) | **Categories E, G**  Nasal carriage isolate then clinical isolate less than four months later from one participant (1028 Client), then clinical isolates from two other participants (1037 Client and 1030 Client) at the same clinic 10 and 12 months later. |
| 2 | ST762  (CC1) | Neg | Pos | 3 | 3  (12) | **Categories C, E**  Groin carriage isolate then clinical isolate (finger wound) from the same participant 14 months later (1063 Client). Another groin carriage isolate from a different participant at the same clinic the same month as 1063 clinical isolate (1083 Client). |

| 3 | ST121 | Pos | Neg | 10 | 1 | **Categories B, D, E, F**  Eight clinical isolates from six different time points within a three month period (groin, arm, blood), one groin carriage isolate from the same period, and one skin lesion (arm) isolate four months later, all from the same participant (1027 Client). |
| --- | --- | --- | --- | --- | --- | --- |
| 4 | ST78 | Neg | Neg | 2 | 0 | **Category B**  Skin lesion isolate and nasal carriage isolate six months later from same participant (1091 Client). |
| 5 | ST5  (CC5) | Pos | Pos | 2 | 0  (0) | **Category D**  Skin lesion isolate and clinical isolate from the same participant from the same site at the same time (1023 Client). |
| 6 | ST93 | Pos | Pos | 6 | 4  (20) | **Category D, F, G**  Two skin lesion isolates (multiple colonies from the same swab) then a clinical isolate from the same participant less than five months later (1078 Client). Three clinical isolates from a second participant within one month (1027 Client). |
| 7 | ST5  (CC5) | Neg | Neg | 8 | 5  (17) | **Categories A, C**  Seven nasal carriage isolates from six different time points over a 16 month period from the same participant (1045 Staff), and one axilla carriage isolate in another participant during that period (1029 Client). |
| 8 | ST72  (CC8) | Neg | Neg | 3 | 3  (6) | **Category C**  One axilla and two groin carriage isolates each from different participants within a six month period (1074 Client, 1076 Client, and 1079 Client). |
| 9 | ST953 | Neg | Neg | 2 | 3 | **Category C**  Two groin carriage isolates from different participants 10 months apart (1025 Client and 1078 Client). |

| 10 | ST1  (CC1) | Neg | Pos | 3 | 5 | **Category C**  Two groin carriage isolates and one nasal carriage isolate each from different participants over a 10 month period (1075 Client, 1079 Client, 1104 Client). |
| --- | --- | --- | --- | --- | --- | --- |
| 11 | ST93 | Pos | Pos | 4 | 0  (0) | **Categories B, C**  One axilla carriage isolate and one groin carriage isolate in one participant (1050 Staff), one groin carriage isolate in another participant (1100 Staff), and one vascular access site isolate (1084 Client), all at the same time. |
| 12 | ST762  (CC1) | Neg | Pos | 2 | 0  (1) | **Category B**  Groin carriage isolate and axilla carriage isolate from the same participant three months apart (1079 Client). |
| 13 | ST5 SLV  (CC5) | Neg | Neg | 4 | 3 | **Categories A, B**  Three groin carriage isolates and one nasal carriage isolate from the same participant all at different times over an 11 month period (1049 Staff). |
| 14 | ST5  (CC5) | Neg | Neg | 2 | 3  (13) | **Category B**  Nasal carriage isolate and groin carriage isolate from the same participant 20 months apart (1027 Client). |
| 15 | ST6  (CC5) | Neg | Neg | 3 | 0 | **Category B**  One nasal carriage isolate, one groin isolate, one skin lesion isolate from same participant, at the same time (1081 Client). |
| 16 | ST6  (CC5) | Neg | Neg | 2 | 1 | **Category B**  One nasal carriage isolate and one axilla isolate from same participant four months apart (1074 Client). |
| 17 | ST508  (CC45) | Neg | Neg | 2 | 0 | **Category B**  One nasal carriage isolate and one axilla isolate from the same participant at the same time (1099 Client). |
| 18 | ST88 | Neg | Neg | 3 | 2 | **Categories A, B**  Two groin carriage isolates and one nasal carriage isolate in the same participant over a 14 month period (1007 Client). |
| 19 | ST101 | Neg | Neg | 5 | 2 | **Categories A, B**  One nasal carriage isolate, two axillar isolates, and two groin isolates, all from same participant at three different time points over a five month period (1024 Client). |
| 20 | ST789  (CC8) | Neg | Neg | 4 | 2  (5) | **Categories A, B**  Three nasal carriage isolates from three different time points, and one axillar carriage isolate over a six month period (1050 Staff). |
| 21 | ST45  (CC45) | Neg | Neg | 2 | 0 | **Category A**  Two skin lesion isolates from the same participant at the same time (1025 Client). |
| 22 | ST5  (CC5) | Neg | Neg | 5 | 2  (15) | **Category A**  Five nasal carriage isolates from the same participant at five different time points over a 12 month period (1021 Client). |
| 23 | ST5  (CC5) | Neg | Neg | 2 | 0  (1) | **Category A**  Two nasal carriage isolates from the same participant four months apart (1099 Client). |
| 24 | ST5 SLV  (CC5) | Neg | Neg | 2 | 0 | **Category A**  Two nasal carriage isolates from the same participant two months apart (1100 Staff). |
| 25 | ST1  (CC1) | Neg | Neg | 2 | 1 | **Category A**  Two nasal carriage isolates from the same participant two months apart (1087 Client). |
| 26 | ST15  (CC15) | Neg | Neg | 2 | 1 | **Category A**  Nasal carriage isolates from the same participant seven months apart (1111 Client). |
| 27 | ST15  (CC15) | Neg | Neg | 3 | 2 | **Category A**  Three axillar isolates from the same participant at two different time points three months apart (1079 Client). |
| 28 | ST12 | Neg | Neg | 9 | 1 | **Category A**  Nine nasal isolates from the same participant at eight different time points over an 18 month period (1046 Staff). |
| 29 | ST72  (CC8) | Neg | Neg | 5 | 0  (3) | **Category A**  Five nasal carriage isolates from the same participant from three different time points over a four month period (1081 Client). |
| 30 | ST97  (CC97) | Neg | Neg | 2 | 3 | **Category A**  Two groin carriage isolates from the same participant five months apart (1041 Staff). |
| 31 | ST834  (CC1) | Neg | Neg | 3 | 1 | **Category A**  Three nasal carriage isolates from the same participant at three different time points over a 10 month period (1034 Staff). |
| 32 | ST8  (CC8) | Neg | Neg | 2 | 1 | **Category A**  Two nasal carriage isolates from the same participant five months apart (1048 Staff). |
| 33 | ST239  (CC8) | Neg | Pos | 3 | 5 | **Category G**  One clinical isolate from one participant (1032 Client) then two clinical isolates from a second participant at one time point (1037 Client) 14 months later. |
| 34 | ST93 | Pos | Pos | 2 | 0  (1) | **Category F**  Two clinical isolates (one wound, one blood culture) from the same participant within two days (1120 Client). |
| 35 | ST15 | Neg | Neg | 2 | 2 | **Category C**  Two nasal carriage isolates from two client participants (1075 and 1096) six months apart. |

* Maximum number of SNPs between any pair of isolates in the group using the all isolate orthologous SNP matrix; (maximum number of SNPs between any pair of isolates in the group using the ST specific orthologous SNP matrix)
