# Supplementary material for: Longitudinal whole-genome based comparison of carriage and infection associated Staphylococcus aureus in northern Australian dialysis clinics
Source: PLoS One. 2021 Feb 5;16(2):e0245790. doi: 10.1371/journal.pone.0245790 (PMC7864423; doi:10.1371/journal.pone.0245790)
Supplement: S6 Data — C: Client participant; S: Staff participant. (DOCX) [file pone.0245790.s007.docx]

**S6 Data. Unique participant-isolate pairs.**

C: Client participant, S: Staff participant.

1. **Carriage**

| **Participant** | **CC** | **mecA** | **PVL** | **Skin lesion** |
| --- | --- | --- | --- | --- |
| 1001 C | 5 | - | - | - |
| 1003 C | 5 | - | - | - |
| 1004 C | 1 | + | - | - |
| 1006 C | 15 | - | - | - |
| 1007 C | 88 | - | - | - |
| 1007 C | 101 | - | - | - |
| 1008 C | 45 | - | - | + |
| 1010 C | 1 | - | - | + |
| 1010 C | 93 | + | + | - |
| 1015 C | 101 | - | - | - |
| 1021 C | 5 | - | - | - |
| 1022 C | 93 | - | + | - |
| 1023 C | 5 | + | + | + |
| 1024 C | 101 | - | - | - |
| 1025 C | 45 | - | - | + |
| 1025 C | 97 | - | - | - |
| 1025 C | 3628 | - | - | - |
| 1026 C | 8 | - | - | - |
| 1027 C | 5 | - | - | - |
| 1027 C | 121 | - | + | + |
| 1028 C | 1 | + | - | - |
| 1029 C | 5 | - | - | - |
| 1033 C | 15 | - | - | - |
| 1042 C | 1 | + | - | - |
| 1063 C | 1 | + | - | - |
| 1072 C | 97 | - | - | - |
| 1074 C | 5 | - | - | - |
| 1074 C | 8 | - | - | - |
| 1074 C | *S. argenteus* | + | - | - |
| 1075 C | 1 | + | - | - |
| 1075 C | 15 | - | - | - |
| 1076 C | 8 | - | - | - |
| 1077 C | 20 | - | - | - |
| 1078 C | 1 | + | - | + |
| 1078 C | 93 | + | + | + |
| 1078 C | 97 | - | - | - |
| 1078 C | *S. argenteus* | - | - | - |
| 1079 C | 1 | - | - | - |
| 1079 C | 1 | + | - | - |
| 1079 C | 8 | - | - | - |
| 1079 C | 15 | - | - | - |
| 1079 C | 97 | - | - | - |
| 1079 C | *S. argenteus* | - | - | - |
| 1081 C | 5 | - | - | + |
| 1081 C | 8 | - | - | - |
| 1083 C | 1 | + | - | - |
| 1084 C | 93 | + | + | - |
| 1087 C | 1 | - | - | - |
| 1088 C | 1 | + | - | - |
| 1090 C | 5 | - | - | - |
| 1090 C | 239 | + | - | - |
| 1091 C | 8 | - | - | + |
| 1091 C | 78/88 | - | - | + |
| 1096 C | 15 | - | - | - |
| 1098 C | 8 | - | - | - |
| 1099 C | 5 | - | - | - |
| 1099 C | 45 | - | - | - |
| 1111 C | 15 | - | - | - |
| 1034 S | 1 | - | - | - |
| 1041 S | 5 | - | - | - |
| 1041 S | 97 | - | - | - |
| 1045 S | 5 | - | - | - |
| 1046 S | 12 | - | - | - |
| 1048 S | 8 | - | - | - |
| 1049 S | 5 | - | - | - |
| 1049 S | 45 | - | - | - |
| 1050 S | 8 | - | - | - |
| 1050 S | 93 | + | + | - |
| 1051 S | 45 | - | - | - |
| 1068 S | 93 | + | + | - |
| 1089 S | 5 | - | - | - |
| 1100 S | 5 | - | - | - |
| 1100 S | 93 | + | + | - |
| 1104 S | 1 | + | - | - |
| 1104 S | 5 | - | - | - |

1. **Clinical**

| Participant | CC | mecA | PVL |
| --- | --- | --- | --- |
| 1023 C | 5 | + | + |
| 1025 C | 93 | - | + |
| 1027 C | 93 | + | + |
| 1027 C | 121 | - | + |
| 1028 C | 1 | + | - |
| 1030 C | 1 | + | - |
| 1032 C | 239 | + | - |
| 1035 C | 121 | - | + |
| 1037 C | 8 | - | - |
| 1037 C | 239 | + | - |
| 1037 C | 1 | + | - |
| 1063 C | 1 | + | - |
| 1078 C | 93 | + | + |
| 1091 C | 30 | - | - |
| 1091 C | 5 | - | - |
| 1120 C | 93 | + | + |
